# Recent Progress in Electrospun Polyacrylonitrile Nanofiber-Based Wound Dressing

**DOI:** 10.3390/polym14163266

**Published:** 2022-08-11

**Authors:** Chang Huang, Xizi Xu, Junhao Fu, Deng-Guang Yu, Yanbo Liu

**Affiliations:** 1 School of Materials and Chemistry, University of Shanghai for Science and Technology, Shanghai 200093, China; 2 School of Textile Science and Engineering, Wuhan Textile University, Wuhan 430200, China

**Keywords:** wound dressings, nanofibers, electrospinning, PAN, wound healing

## Abstract

Bleeding control plays a very important role in worldwide healthcare, which also promotes research and development of wound dressings. The wound healing process involves four stages of hemostasis, inflammation, proliferation and remodeling, which is a complex process, and wound dressings play a huge role in it. Electrospinning technology is simple to operate. Electrospun nanofibers have a high specific surface area, high porosity, high oxygen permeability, and excellent mechanical properties, which show great utilization value in the manufacture of wound dressings. As one of the most popular reactive and functional synthetic polymers, polyacrylonitrile (PAN) is frequently explored to create nanofibers for a wide variety of applications. In recent years, researchers have invested in the application of PAN nanofibers in wound dressings. Research on spun nanofibers is reviewed, and future development directions and prospects of electrospun PAN nanofibers for wound dressings are proposed.

## 1. Introduction

Skin is the largest organ of the human body: it is able to maintain fluid balance, regulate body temperature, resist the invasion of pathogens and microorganisms, prevent microbial infection, resist the invasion of various organs and tissues by harmful factors in the external environment, prevent all kinds of nutrients within the organization and the loss of water, electrolyte and other substances; with its sensory functions, skin has an extremely important role [1,2,3,4]. At the same time, the skin is also the largest part of the human body in contact with the external environment. There is a wide variety of bacteria on the skin surface, including staphylococcus, streptococcus, *Candida albicans (C. albicans)*, and non-pathogenic mycobacteria. When the skin is damaged, it is vulnerable to infection from the external environment. Bacteria will accumulate around the wound, resulting in bacterial infection, causing tissue dehydration, and even leading to serious secondary trauma [5,6].

As a barrier to direct contact with the internal and external environment, the skin is in a state of long-term exposure, which makes skin damage inevitable [7]. If some non-self-healing wounds are not treated in time, a large amount of extracellular fluid will be lost in the wound, and in severe cases it will be life-threatening [8,9,10]. Based on the above situation, this promotes the research and development of hemostatic wound dressings. Considering the complexity of the wound healing process, good biocompatibility, stable structure, and good mechanical properties are all necessary for an ideal wound dressing [11,12]. In addition, the wound dressing should be able to absorb the excess biological fluid in the wound in time, protect the microenvironment of the wound from contamination, and keep the area around the wound moist it should also have good air permeability and be able to function in the process of wound healing to promote cell growth and accelerate wound healing [13].

In fact, the initial wound dressings, including gauze and bandages, can only be used to protect the wound from external stimulation. Gauze, mostly made of nonwoven fabric, absorbs exudates and fluids from an open wound, and it is used to clean and dry the wound. Most bandages are made of natural cotton, wool, cellulose, rayon, and polyester [14]. However, cotton bandages will adhere to some fibers on the surface of the wound when cleaning the wound, which is unfavorable for the treatment of the wound. Later improved rayon and polyester bandages do not shed fibers on the wound surface and can absorb exudates to some extent [14], but there are still problems that traditional wound dressings cannot solve [15]: (1) Poor adhesion, unable to provide adequate drainage for the wound; (2) Difficulty maintaining the wetness of the wound, the dressing easily sticks to granulation tissue and is not easy to clean; (3) It is relatively difficult to treat a wound of a special position or a special shape and has no significant effect; (4) The dressing needs to be changed regularly, which may cause secondary injury to the wound and delay the healing time of the wound; (5) Failure to control drug release intended for accelerated wound healing.

With the development and iteration of wound dressings, its appearance is also varied. Statistics show that wound dressings currently on the market exist in different forms such as hydrogels [16,17,18,19], films [20,21,22,23], sponges [24,25,26], scaffold [27,28,29,30,31,32,33], and nanofibers [34,35,36,37]. Nanofibers have good specific surface area, porosity and mechanical properties, which are not only easy to manufacture, but also easy to surface modification. Thus, in turn, they have a wide variety of potential applications owing to their unique advantages [38,39]. In recent years, nanofibers have been increasingly applied in biomedicine (including wound dressing and drug delivery [40,41], tissue engineering [42], biosensing, regenerative medicine), environmental applications [43,44,45,46], catalysis [47,48], and electronic information [49,50,51,52]. Nanofiber fabrication techniques [53] include bottom-up and top-down methods such as stretching [54], template synthesis [55], self-assembly [56], microphase separation [57], electrospinning [58], dry spinning [59], wet spinning [60], melt spinning [61], solution blow spinning [62], centrifugal spinning [63], and microfluid spinning [64].

As one of the most economical and efficient methods to produce nanofibers, electrospinning is gaining popularity. As more and more related publications have reported on it, it has also received a lot of attention. Electrospinning is a bottom-up method of nanofiber synthesis, which is unique in the biomedical field [58]. Compared with other fibers, the internal pore structure of electrospun nanofibers can be controlled [65,66], and the fiber composition and structure can be changed according to performance requirements [67]. Its high porosity provides a more adequate contact surface for gas exchange and liquid absorption [68], resulting in excellent permeability, which keeps the wound moist when used as a wound dressing and acts as a barrier against microbial invasion [69,70,71]. At the same time, the high flexibility of the nanofiber itself makes the wound dressing suitable for different parts and different shapes of the wound, promoting wound healing and possibly having a certain potential of anti-scar formation [72,73].

In electrospinning technology, single axial is one of the simplest and most basic technologies. Through different collectors and post-processing techniques, the structure of the nanofibers is changed [74]. The molecular structure can also be controlled by the length of the needle tube to achieve self-assembly of large molecules [75]. Of course, on a single-axial basis, the research of multi-axial electrospinning technology can better meet more special needs. Electrospinning and interface assembly techniques are mainly used to synthesize fibers of different structures, and fibers with irregular cross-sections can be synthesized by changing the shape of the nozzle [76]. As we know, many soft tissues in the human body have mechanical anisotropy, and we can also control the alignment and morphology of fibers by designing electrospinning collectors to simulate the reproduction of human soft tissue structure and build anisotropic scaffolds [77]. It is also possible to use the isotropic homogeneity of traditional electrospinning nanofibers to form a fiber film with different fiber arrangements by combining electrospinning and coaxial electrospinning [78].

**Figure 1:**
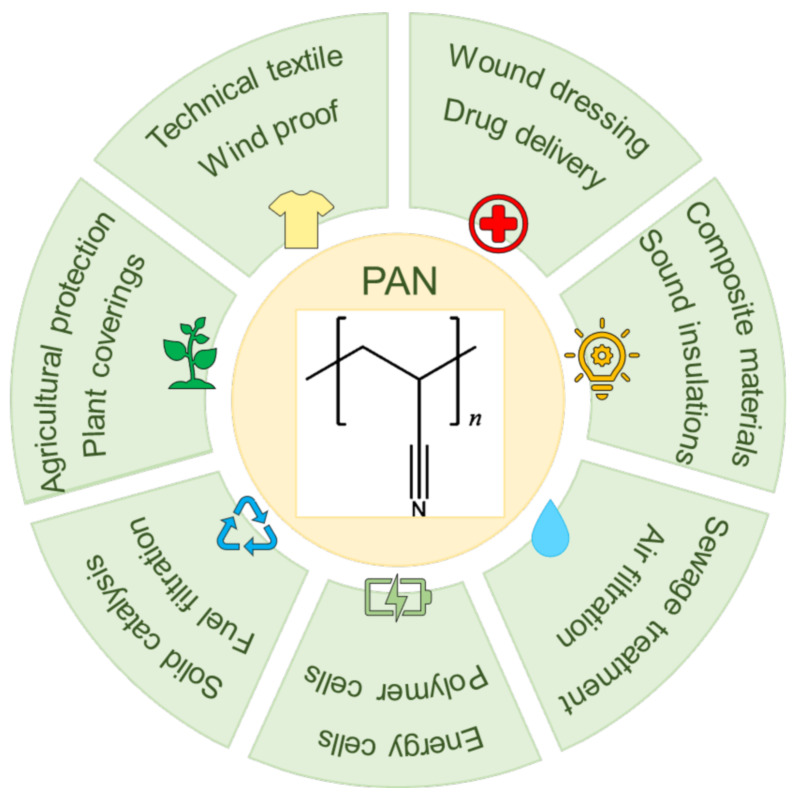
Applications of PAN.

PAN is a white semi-crystalline synthetic organic polymer, first manufactured by Rain in Germany in 1931. However, the polymer is insoluble in most organic and inorganic solvents, so researchers did not make PAN fibers until the discovery of the dimethylformamide solvent. As a thermoplastic polymer, PAN has good chemical resistance and excellent mechanical properties, and it is an important raw material for ultrafiltration membranes, fabric fibers, carbon fibers, reverse osmosis hollow fibers, and so on [79]. The produced PAN fibers have also been used in heavy metal adsorption [80,81,82], aerospace technology [83], solid catalysis [84], wound dressings [85], etc. Its common applications are shown in Figure 1.

In this review, the four processes of wound healing and the existing wound care systems are described in detail. Based on the existing electrospinning technology and existing research, the properties of PAN nanofibers were analyzed, and the modification technology of PAN nanofibers was introduced. On this basis, the current application and development status of PAN-based electrospun fibers in wound dressings are expounded. According to the current medical demand for wound dressings, the problems and challenges faced by the creation and research and development of new wound dressings at this stage are pointed out, and the development direction and prospect of PAN nanofibers for wound dressings in the future are proposed on the basis of summarizing the existing improved PAN electrospun fibers.

## 2. Wound Healing Process and Ideal Wound Care System

A wound is a disruption of normal anatomical structure and function when the skin is physically, chemically, and thermally damaged [86]. Wounds can be divided into acute wounds and chronic wounds according to their healing time [87]. Acute wounds generally heal completely within two to three months, while chronic wounds begin to heal after three months [88].

### 2.1. Wound Healing Process

**Figure 2:**
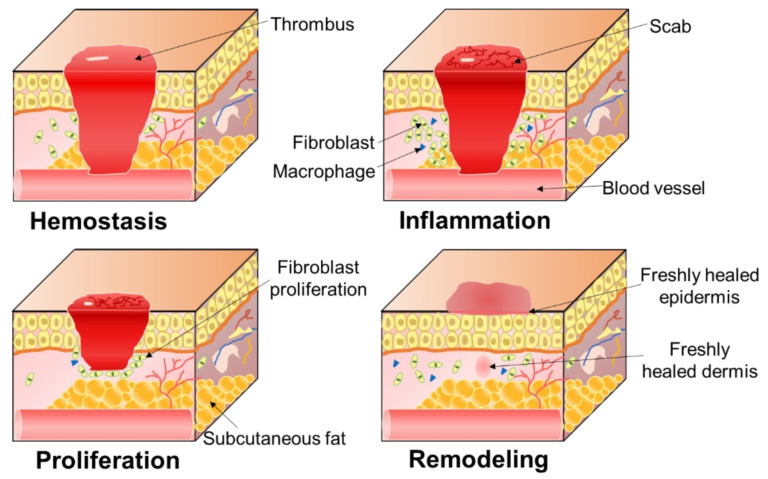
Wound healing process.

Wound healing involves four distinct stages (Figure 2): hemostasis, inflammation, proliferation, and remodeling, and these four stages overlap in time.

#### 2.1.1. Hemostasis

Hemostasis is the first stage of wound healing and is an immediate response triggered to prevent blood loss after detection of microvascular damage. It usually lasts 1–3 h [89]. Hemostasis is a strictly regulated process of blood coagulation, platelet activation, and vascular repair [90]. First, vasoconstriction occurs at the injury site, causing platelet aggregation and activation at the injury site, forming a platelet thrombi and reducing blood flow [91,92]. In the process of coagulation cascade, fibrinogen thrombin lysates around platelet thrombus to generate cross-linked fibrin network, forming stable clots; meanwhile, cytokines and protein factors released by activated platelets serve as the reservoir of the formed clots, acting on the wound healing process [93,94,95].

#### 2.1.2. Inflammation

Inflammation is the second stage of wound healing and usually lasts approximately 24–72 h. The inflammatory phase is a process of controlling bleeding and creating a clean wound, and lymphocytes release vasoactive factors into diastolic blood vessels to increase capillary permeability. After wound bleeding is controlled, neutrophils formed from adjacent blood vessel damage will appear at the injury site to engulf bacteria and cellular debris. Finally, monocytes penetrate into the wound and are activated to become macrophages, which remove apoptotic cells, damaged extracellular matrix, and debris and bacteria, and they restore damaged tissue, participating in host defense mechanisms [96,97,98].

#### 2.1.3. Proliferation

Proliferation is the third stage of wound healing and can last up to 20 days. The proliferative stage is the connective tissue hyperplasia stage, which is the process of blood vessel formation, tissue granulation (including the presence and proliferation of fibroblasts, keratinocytes, endothelial cells, and thin-walled capillaries), and re-epithelialization [94]. At this stage, endothelial cells venules protrude into the wound and extracellular matrix to form a network of capillaries and arterioles [97], new blood vessels are generated from existing vessels, and both fibroblasts and keratinocytes can produce TGF-β (TGF-β), inducing the formation of granulation tissue and the differentiation of myofibroblasts and the migration of epithelial cells from the wound edge to accelerate wound closure [99].

#### 2.1.4. Remodeling

Remodeling is the final stage of wound healing, during which the structural collagen network of healthy tissue is restored [89]. Fibroblasts regulate the decomposition of wound matrix by synthesizing matrix metalloproteinases and new extracellular matrix, promoting wound contraction and alleviating epithelialization and scar formation [93,97]. This phase can even last for a year or two.

### 2.2. Ideal Wound Care System

In daily life, thousands of people all over the world are injured every day due to burns, abrasions, cuts, and so on, and the degree of skin damage at these wounds varies [100]. Skin lesions in acute wounds are relatively small and shallow, mainly mechanical injuries such as abrasions and cuts. Acute wounds will have epidermal damage and oozing, and acute wounds with mild injuries can heal spontaneously [86]. Chronic wounds heal slowly and are prone to scarring. Repeated tissue damage, infection, thermal damage, and physiological diseases may form chronic wounds [88]. Macrophages and neutrophils are damaged in chronic wounds, the inflammation phase is prolonged, infection is prone to occur, and the healing process is disordered [101].

For a healthy patient, the ideal goal of wound care therapy is to [102]: (1) Protecting the wound from bacterial infection, mechanical stress and other external factors that slow wound healing; (2) Keeping the wound moist to accelerate wound healing; (3) Minimizing or avoiding scar formation.

In addition to these basic objectives of wound care, therapeutic care for some chronic refractory wounds should include [102]: (1) Regulating inflammation and stimulating the inflammatory stage of wound healing; (2) Promoting epithelial migration to form granulation tissue through collagen deposition and extracellular matrix remodeling in order to promote the generation of new blood vessels, stimulate tissue blood perfusion and lymphatic angiogenesis, and complete the healing repair stage [103,104]; and (3) Removing necrotic tissue.

In the process of wound nursing, the existence of wound dressing plays a crucial role. To be an efficient wound dressing, the ability to maintain the moisture and gas exchange at the physiological wound is a key feature, which requires that the wound dressings cover the wound, ensure proper moisture content in the body, allow oxygen to grow tissue oxygen permeability, and prevent the growth of pathogens in the environment as much as possible, without interfering with wound healing [105,106]. Simulating natural epithelial cells as a barrier to the invasion of harmful viruses and bacteria in the environment is our goal to improve and to develop wound dressings. The idea of one treatment for all in wound care is outdated, which requires us to be able to select different materials and wound dressings that are appropriate for different wound sites, shapes, and characteristics. Furthermore, wound dressings should be made of materials that are immunocompatible and should not support intracellular growth and cell adhesion, while minimizing the possibility of secondary injury and complications resulting from removal [107,108,109]. To facilitate the wound healing process, the wound dressing should be optimized to control bleeding, maintain antimicrobial activity, maintain the activity of the drug delivered [110], and release the drug at a rate (fast release or slow release) depending on the healing of the wound [12,111]. Finally, the management of exudate is also an important factor to be considered when selecting wound dressings. Factors suitable for bacterial growth such as inflammatory cytokines and chemokines are abundant in wound exudate. It is also particularly important for us to effectively remove wound exudate and not create favorable conditions for bacterial growth under the condition of ensuring that the wound tissue is not dehydrated in a moist environment [11,112].

Wound dressing is one of the important components of wound care, and dressings with different therapeutic effects should be selected according to the damage of different wounds.

## 3. Electrospun Nanofibers for Wound Dressing

### 3.1. Electrospinning Technology

**Figure 3:**
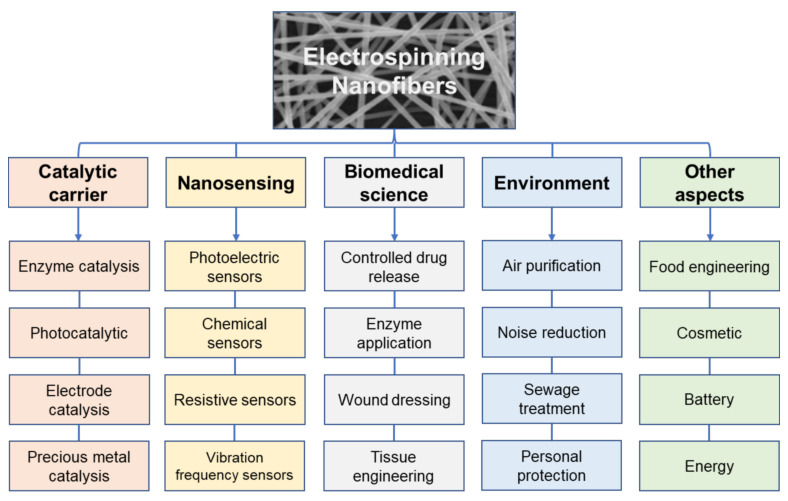
Applications of Electrospinning Nanofibers.

Electrospinning is a simple, efficient technique for producing nanofibers using a small amount of precursor polymers under an electric field [113,114]. Electrospun nanofibers have potential applications in almost all scientific fields, some examples are included in Figure 3.

**Figure 4:**
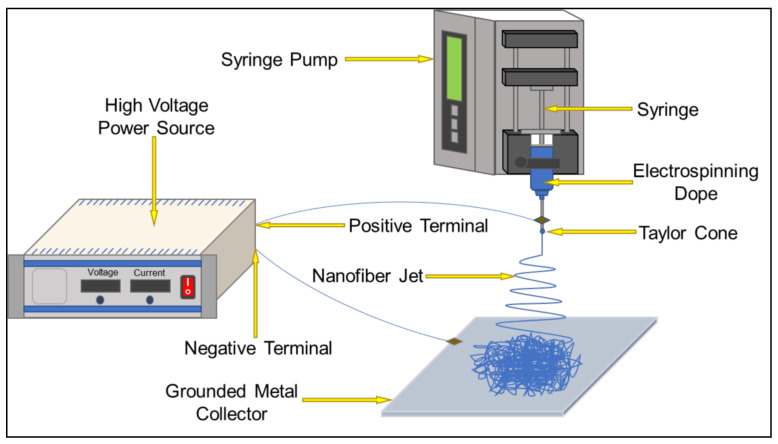
Schematic diagram of the electrospinning device.

Electrospinning equipment consists of three parts: high voltage power supply, spinneret section (including propulsion pump and polymer solution supply unit), and receiving unit [115,116], as shown in Figure 4. Under a constant high-voltage power field, a syringe filled with polymer solution is placed in the field, and the nozzle of the syringe is driven by the high-voltage electric field to push the solution to spray continuously. When the applied high voltage is enough to overcome the surface tension of the solution at the nozzle, a “Taylor cone” will be formed at the tip of the needle, and the voltage will continue to increase. When the electric field is large enough, the solution is ejected in a trickle, evaporates and solidifies during the ejection process, and it is then deposited on a collection device to form a nonwoven fiber mat, resulting in consistent and uniform nanofibers [117,118,119,120]. The diameter of nanofibers obtained by this technology can reach several hundred nanometers or even less than one hundred nanometers.

During the electrospinning process, the morphological structure of fibers is affected by multiple factors. The properties of the polymer itself (including the relative molecular mass of the polymer, the properties of the solvent, the concentration and viscosity of the spinning solution, the conductivity of the solution, the surface tension of the polymer solution), the electrospinning process parameters (such as potential, flow rate, applied voltage, receiving distance, and the structure and specifications of the spinning head), environmental parameters (such as temperature and humidity) are the most important factors [121,122,123]. These factors are interrelated and synergistic to regulate the shape, diameter, and quality of electrospun fibers and to promote the diversification of nanofibers’ morphology, structure, and properties [124].

Under the premise that many factors affect the morphological structure of nanofibers, with the rapid and widespread use of electrospinning technology, researchers are also constantly exploring ways to predict the diameter of nanofibers according to the properties of spinning fluids and the operating conditions of electrospinning to produce uniform fibers [125]. It has turned out that the fiber diameter has a good correlation with the dimensionless number, and the diameter of the fiber has a clear relationship with the viscosity of the solution [126].

### 3.2. Type and Structure of Electrospun Nanofibers

**Figure 5:**
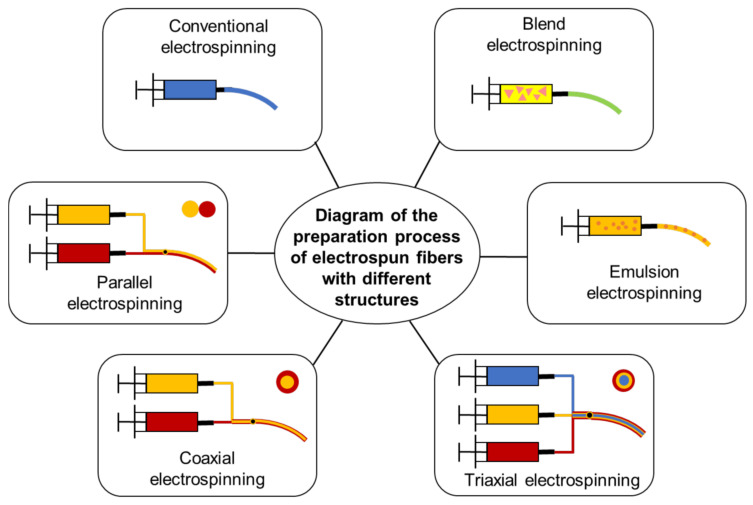
Schematic diagram of the process of electrospun fibers with different structures prepared under corresponding measures.

According to the chemical composition of nanofibers, electrospun nanofibers can be divided into inorganic nanofibers, organic nanofibers, and inorganic organic hybrid nanofibers [127]. In order to regulate the properties of electrospun nanofibers, on the basis of traditional electrospinning technology, modified electrospinning processes, such as hybrid electrospinning [128], emulsion electrospinning [129,130], coaxial electrospinning [131,132,133,134], parallel electrospinning [135,136], and triaxial electrospinning [137,138], have been successively developed, as shown in Figure 5.

The properties of nanofibers vary due to different nanofiber structures, and the choice of functionalized nanofiber structure will also be determined by the application of nanofibers in different fields [139]. In terms of the structure and shape of nanofibers, electrospun nanofibers commonly used in wound dressings include porous nanofibers [140], parallel nanofibers [135], double-layer and triple-layer core-shell nanofibers [141], hollow nanofibers [55], and porous core-shell nanofibers [142].

Due to the high specific surface area and high porosity of electrospun fibers, they show unique advantages in the development of wound dressings. The polymer species currently available in nanofiber wound dressings include natural polymers and synthetic polymers. Natural biomaterials are pretreated before spinning, the regulation of shape and size is relatively complicated, and it is easy to form strong hydrogen bonds with the aqueous solution, which makes the solution viscosity high, which is not conducive to spinning. In comparison, synthesizing polymers is much easier. In practical applications, the selection of nanofiber materials and the regulation of structure need to be considered in combination with specific situations.

## 4. Electrospun PAN Nanofiber

### 4.1. Natural Derivatives and Synthetic Additives of PAN Electrospun Fiber Systems for Wound Dressings

PAN is a polymer compound obtained by radical polymerization of monomer acrylonitrile, and its chemical formula is (C_3_H_3_N)_n_. Electrospinning technology combines the excellent properties of PAN with the unique structural morphology of electrospun fibers to create more considerable value. PAN is soluble in polar organic solvents, and N,N-dimethylformamide (DMF), N,N-dimethylacetamide (DMAc), and dimethyl sulfoxide (DMSO) are often used as benign solvents for PAN as its spinning solvent. In addition, ethylene carbonate, sodium thiocyanate, nitric acid, and zinc chloride can also be used as spinning solvents.

**Figure 6:**
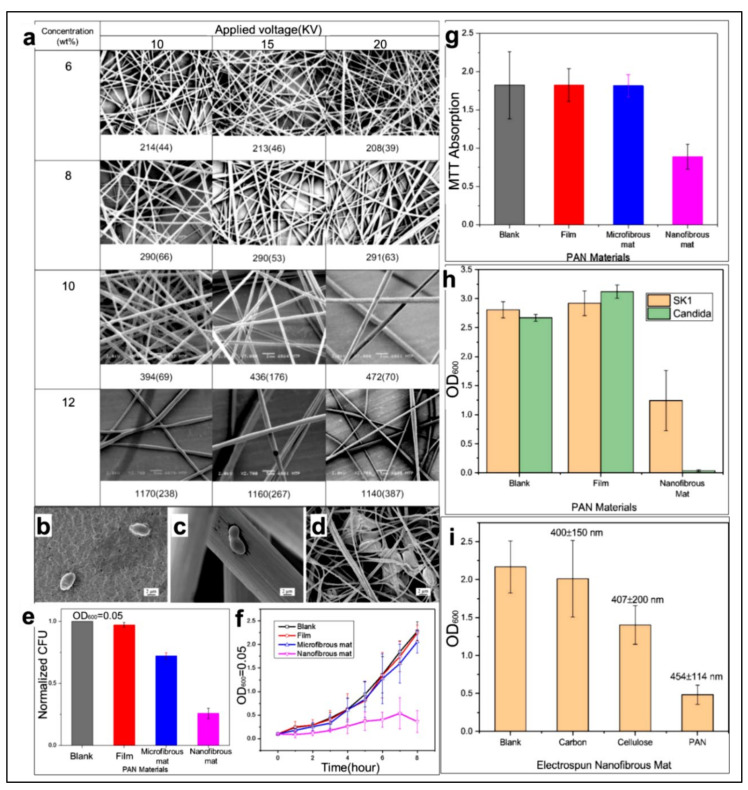
Excellent performance analysis diagram of PAN. (**a**) When the external voltage is 10, 15, 20 KV, and the distance between the spinning head and the aluminum foil receiver is 10 cm, morphology of PAN electrospun nanofibers at concentrations of 6, 8, 10, and 12 wt% with mean fiber diameter (nm) and standard deviation of fiber diameter, Reprinted with permission from [143] copyright 2005, Elsevier. (**b**–**d**) scanning electron microscope images of Saccharomyces cerevisiae SK1 cells after 30 min incubation on PAN films, PAN microfiber mats, and PAN nanofiber mats, respectively; (**e**) Changes in colony forming units of the blank group, PAN film, PAN microfiber mat, and PAN nanofiber mat after exposure to SK1 cultures for 1 h, respectively; (**f**) Growth of SK1 cells cultured in blank group, PAN film, PAN microfiber pad, and PAN nanofiber pad within 0–8 h, respectively; (**g**) Activity of SK1 cells cultured in blank group, PAN film, PAN microfiber pad and PAN nanofiber pad for 8 h, respectively; (**h**) Cell viability of SK1 and *C. albicans* cultured in blank group, PAN film and PAN nanofiber pad for 18 h, respectively; (**i**) SK1 cell activity after culturing for 18 h in blank group, carbon nanofibers with an average diameter of 400 ± 150 nm, cellulose nanofibers with an average diameter of 407 ± 200 nm, and PAN nanofibers with an average diameter of 454 ± 114 nm, respectively, Reprinted with permission from [146] copyright 2019, Elsevier.

PAN has excellent filamentation properties, and the PAN nanofibers prepared by electrospinning are generally small in diameter and uniform in distribution. Gu et al. [143] studied the morphology, average fiber diameter, and diameter distribution of PAN nanofibers with a concentration of 6–12 wt% using DMF as a solvent and external voltages of 10 KV, 15 KV, and 20 KV, respectively (as shown in Figure 6a). With different external voltages, when the concentration is 8 wt% and 10 wt%, uniform fibers with diameters of 200–500 nm can be obtained, and the average diameter of the fibers does not change much with the change of voltage. There are also many studies that can prove the excellent mechanical properties, good thermal stability, and strong weather resistance of PAN nanofibers [144,145]. Sirelkhatim et al. [146] also found lower cell viability of Saccharomyces cerevisiae and *C. albicans* in PAN electrospun nanofibers (among them, *C. albicans* had a very low cell viability after culture in PAN nanofiber mats), showing that PAN electrospun nanofibers have potential antifungal properties (as shown in Figure 6b–i). These excellent properties determine the wide application of PAN nanofibers. Unfortunately, pure PAN electrospun fiber membranes are not sufficiently hydrophilic, and their surfaces are relatively inactive and hydrophobic.

**Figure 7:**
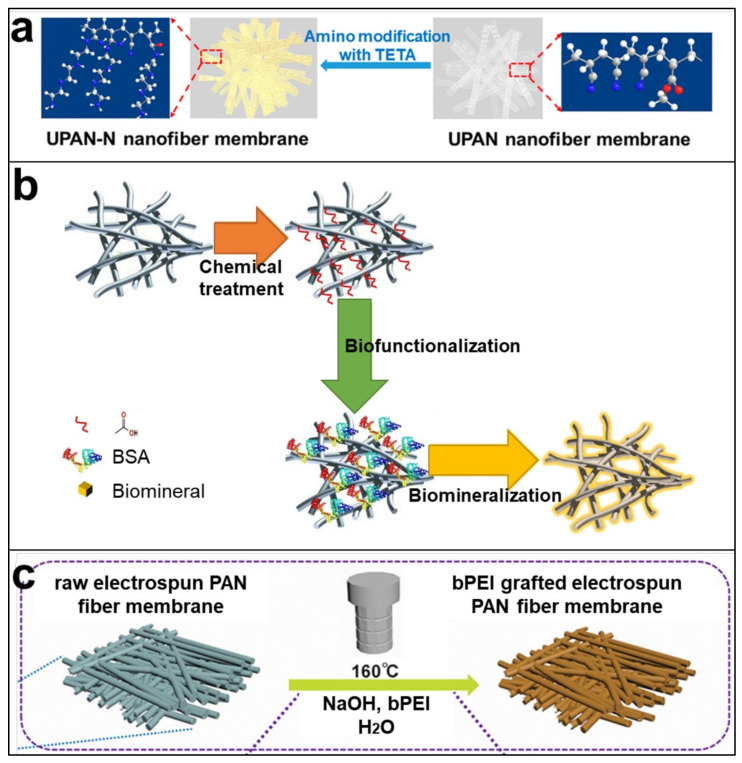
Schematic diagram of the three methods of PAN nanofiber surface modification. (**a**) Schematic of the process of chemically modifying the surface of PAN nanofibers with TETA, Reprinted with permission from [150], copyright 2022, American Chemical Society. (**b**) Schematic diagram of the biomineralization process of PAN nanofibers, Reprinted with permission from [156] copyright 2020, Elsevier. (**c**) Schematic diagram of the whole process of preparing bPEI grafted PAN nanofibers by electrospinning technology and green hydrothermal reaction, Reprinted with permission from [160] copyright 2018, Elsevier.

To improve the hydrophilicity of PAN nanofiber membranes, surface modification methods have been extensively explored to make them hydrophilic due to changes in the surface structure. As a matrix material, PAN is easily modified [147,148], and bringing functional groups such as hydroxyl, amine, imine, and carboxylic acid to the surface of PAN nanofibers through reactions such as reduction, amination, amidation, and hydrolysis by chemical treatment are the most commonly used methods to enhance the surface activity and hydrophilicity of PAN electrospun fiber membranes, allowing these hydrophilic functionalities to exist stably on the membrane surface [149]. Zhao et al. [150] used triethylenetetramine (TETA) to make the surface of PAN nanofibers with a high density of amine groups(as shown in Figure 7a), and the water contact angle test confirmed the increased hydrophilicity of the aminated PAN nanofibers. Huang et al. [151] obtained amidoxime PAN nanofibers with a higher surface activity by reduction reaction of hydroxylamine hydrochloride aqueous solution with PAN nanofibers. As mentioned above, more studies have shown that reactive nitrile groups in PAN can generate hydrophilic groups on the surface of PAN nanofibers through different chemical reactions to achieve the expected modified PAN nanofibers with optimized properties [152,153]. In these chemical treatment processes, alkaline solutions are more relatively used; at the same time, they are widely used due to their relatively simple operation [149]. As we all know, biomacromolecules are nontoxic and can regulate mineral nucleation and growth when used in biomineral-polymer composite biomaterials [154]. Biomineralization is a process in which organisms generate inorganic minerals through the regulation of biological macromolecules. Later, the natural mineralization process has begun to be put into engineering, which can be used in the biomedical field by constructing bio/organic–inorganic hybrid composites with nanostructures [155]; modification of PAN nanofibers by this method can impart the functionality of their inorganic components, while retaining the biocompatibility of biopolymers. Homaeigohar et al. [156] explorably developed a bovine serum albumin (BSA)/PAN biohybrid nanofiber wound dressing. In this study, PAN nanofibers were functionalized with an inexpensive biomacromolecule BSA after chemical post-treatment, resulting in biomineralized nanofibers. The mineralization process is shown in Figure 7b. Obviously, the biomineralized PAN nanofibers have elastic modulus similar to that of human skin, and they show a higher cell viability for fibroblasts and keratinocytes, which can promote the generation of granulation tissue in the middle and late stages of wound healing. At the same time, this method has a low processing cost and can be directly processed, which needs to be further developed to achieve the purpose of large-scale production of nanofibers with excellent biological activity and mechanical properties.

In addition to the above two methods, methods such as plasma treatment [157], surface grafting of hydrophilic polymers [158], and coatings are also widely used. The abundant active nitrile groups in PAN also create opportunities for physical or chemical grafting on the surface of PAN nanofibers [159]. Chemical grafting is more stable than physical grafting, so there are more applications for related research. Zhao et al. [160] took advantage of the biocompatibility and high hydrophilicity of polyelectrolyte branched polyaniline (bPEI) of polyelectrolytes to graft them onto the PAN nanofibers through green hydrothermal reaction(as shown in Figure 7c), giving a certain density of amine groups on the surface of PAN nanofibers, and significantly reducing the water contact angle of the fiber membrane.

### 4.2. Electrospun Fibers Blended with PAN and Other Polymers

Adding another polymer to PAN for blend electrospinning can solve the shortcomings of single polymer spinning to a certain extent. By blending electrospinning, not only the hydrophobicity of PAN but also the spinnability of hydrophilic polymers can be improved. These polymer materials can be mainly divided into two categories: natural polymers and synthetic polymers. Commonly used natural polymers include sodium alginate [161,162,163] (SA), chitosan [164,165,166] (CS), gelatin [167,168,169] (GEL), and hyaluronic acid [34,170,171] (HA). Commonly used synthetic polymers include polyvinyl alcohol (PVA), polyacrylamide (PAM), polyethylene glycol (PEG), polymethylmethacrylate (PMMA), PAN, polycaprolactone (PCL), Polyurethane (PU), and so on. Some of the polymers used for electrospinning in blends with PAN are listed in Table 1.

Amino-rich polyaniline (PANI) is hydrophilic, and Shakiba et al. [173] prepared blend nanofiber membranes with PANI (10, 20, 30 and 40 wt%) at temperatures of 25, 40, and 55 °C, respectively. At 40 °C, the structure of PAN/40 wt% PANI nanofiber was uniform, and the surface of PAN/40 wt%PANI@40 °C nanofiber membrane was rougher and more hydrophilic than the pure PAN nanofiber membrane. It also showed better mechanical properties in the tensile test, and its pore size was similar than that of the pure PAN nanofiber, but the average pore size increased significantly. PAN/40 wt%PANI@40 °C nanofiber can well improve the problem of insufficient hydrophilicity of pure PAN nanofiber on the premise of retaining the excellent properties of pure PAN nanofiber.

## 5. Research Progress of PAN-Based Electrospun Fibers in the Development of Wound Dressings

In order to enable wound dressings to achieve the ideal effect of providing a moist microenvironment, promoting epithelialization and cell migration into the wound, exhibiting mechanical stability during application, and acting as a barrier against external threats, such as microorganisms or tissue destructive forces, materials should also be certainly chosen for preparing wound dressings. The optimal material should ensure permeability to gases and fluids and avoid the formation of necrotic tissue due to dehydration and accumulation of exudates [183]. To date, a large number of polymeric materials have been developed for the preparation of wound dressings for different clinical treatment regimens [184]. Natural polymer nanofibers have biodegradability and compatibility, which once attracted the attention of many researchers. However, it cannot be ignored that the high hydrophilicity and poor mechanical properties of natural polymers are also the reasons why such dressings are prone to deformation. In addition, its processing performance is poor and the price is expensive, which is not conducive to mass production. This problem has not been well resolved so far.

The high molecular orientation, weather resistance, and good mechanical properties exhibited by PAN [185,186], as well as its advantages in industrialization and large-scale production, make it quite a market for the manufacture of carbon fibers. At the same time, it is also one of the most important materials in the field of biomedicine. Despite this, however, few relatively biomedical articles of this material have been reported [156]. As mentioned above, in order to maintain the properties of PAN itself and obtain high potential for wound dressings, PAN nanofibers can be surface-modified or blended with other polymers to endow PAN nanofibers with new properties. Simultaneously, in order to promote faster wound healing when PAN nanofibers are used in wound dressings, it can be achieved by adding additives to improve performance or changing the structure of PAN electrospun nanofibers. In pharmaceutics, the drugs are frequently added into the polymeric carriers as additives to endow the functional performances of the final products [40,119,187]. In the following, the related research on enhancing the properties of PAN nanofibers and expanding the application of PAN electrospun fibers in wound dressings will be elaborated from three aspects.

### 5.1. Natural Derivatives and Synthetic Additives of PAN Electrospun Fiber Systems for Wound Dressings

**Figure 8:**
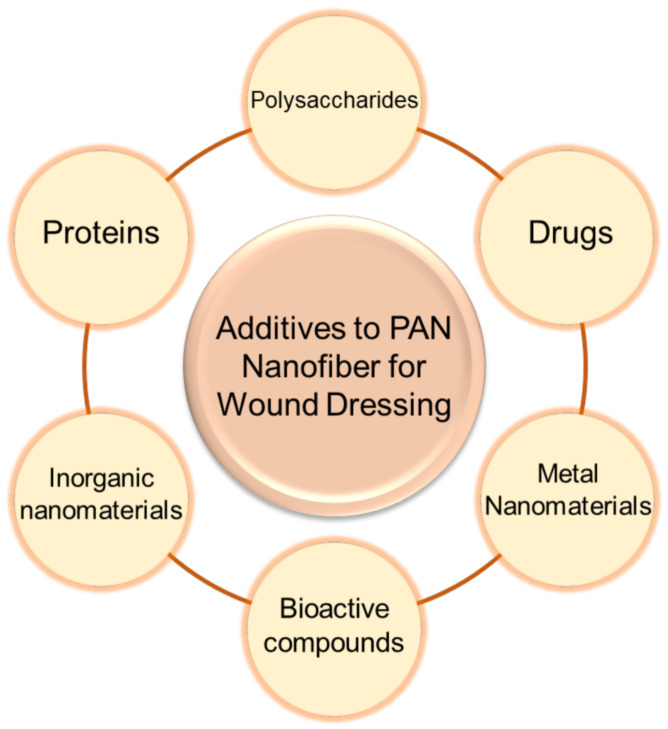
Various additives used in PAN nanofibers to enhance their biological and physicochemical properties as wound dressings.

In order to extend the applicability of the PAN nanofiber wound dressing, the studies of PAN mixed natural derivatives and other substances that can promote wound healing have increased. The various additives for the PAN nanofibers to enhance their biological and physical chemical properties as a wound dressing are summarized in Figure 8.

#### 5.1.1. Drug Load with Wound Healing Properties

Wound dressings can be divided into passive dressings, interactive dressings, and bioactive dressings [188,189,190]. In order to cooperate with the rapid healing of wounds, some therapeutic drugs can be added to the nanofibers [191]. The use of drugs in PAN drug-loaded nanofiber wound dressings over the past decade is summarized in Table 2.

When PAN electrospun fibers are directly used as wound dressings, they can only be used as passive dressings to cover the wound surface, absorb exudates, block the invasion of external bacteria, and provide limited protection for the wound surface, but they cannot have other antibacterial and anti-inflammatory effects [199,200]. In the process of wound healing, it is usually necessary to use a certain drug to promote anti-inflammation and to accelerate the speed of wound healing. Among them, oral medication is the most commonly used method. However, when this method of oral administration is used, the action of the drug is relatively slow and irregular; the absorption and utilization of the drug will be affected by the gastrointestinal function, the speed of gastric emptying, and the concentration of gastric acid and other factors; it will bring certain stomach irritation; and some of the drug will be destroyed and invalid if taken orally. In addition, there is also a point that cannot be ignored, which is the use of drug is affected by the state of the patient. If the patient is in a state of unconsciousness or coma, it is not suitable for oral administration. On this basis, in order to improve the side effects of oral administration of a drug, researchers use transdermal delivery as an alternative to oral administration [201,202]. This improved local drug delivery with a controlled rate is superior to systemic drug administration, enhances wound healing, reduces toxicity, and has certain applicability. Sarwar et al. [192] used nanoencapsulation technology to load the non-steroidal anti-inflammatory drug diclofenac (DLF) into PAN nanofibers to evaluate the applicability of this drug-loaded nanofiber for transdermal absorption therapy. Encapsulating DLF in PAN fibers can not only retain the anti-inflammatory and analgesic properties of DLF but also have the possibility to reduce the toxicity of DLF itself and the harm caused by other adverse reactions, avoiding the degradation of DLF in vivo caused by oral administration. The DLF-encapsulated PAN nanofibers also maintained long-term bioactivity during the infiltration, diffusion, and slow release of DLF from the fiber surface until DLF was inactivated. When nano-encapsulation technology is used for drug release, drug and other biologically active substances can be coated in nanofibers. This technology is easy to operate, achieves local delivery of drugs while reducing drug toxicity and side effects, controls the drug release profile to a certain extent, and brings many clinical applications for transdermal drug delivery. Bioactive substances extracted from plants can also be used as medicines, can sustainably release growth factors required for wound healing when used in wound dressings, and are suitable for burns. Fayemi et al. [196] used plant extracts to study a PAN nanofiber containing different concentrations of Moringa oleifera leaf extract, its antibacterial activity increased with the concentration of Moringa oleifera leaf extract in the fiber. The wound-healing properties were explored in mouse experiments, and the results showed that the speed of wound closure was consistent with its antibacterial activity. In addition, there are other drugs loaded into PAN nanofibers that endow them with different effects, so that the drug-loaded PAN nanofibers can be used as dressings for wounds with different diseases [194,196]. In the transdermal delivery of drugs, it is worth noting that the solubility of the drug in the polymer system plays a decisive role in the drug distribution in the nanofibrous membrane, affecting its bioavailability and penetration [203,204,205,206,207].

As an organic fiber, PAN nanofiber has a relatively smooth surface. When drug molecules are loaded, they do not easily adhere, and it is easy to burst and release at the wound. In order to increase the drug-loading and drug-releasing capacity of the fibers and to reduce the frequency of dressing replacement, it is necessary to modify the fiber surface. Metal-organic framework (MOF) has porous structures with a high specific surface area and a controllable pore size [208], making them one of the most promising drug delivery carriers [209]. To effectively control the drug release rate, Yang et al. [210] embedded gentamicin-loaded ZIF-8@gentamicin into PAN/GEL nanofibers. It has been proven that the larger specific surface area of ZIF-8 increases the drug loading, and the drug release rate used in wound dressings also changes with the pH value of the wound environment, effectively shortening the wound healing time. The results of in vivo wound experiments in rats also confirmed that the effect of composite nanofibers on wound recovery is greater than that of single-spind nanofiber membranes than traditional gauze.

#### 5.1.2. Other Additives with Wound Healing Properties

Nanofibers can not only construct drug transdermal delivery systems, but also add other functional particles, such as antibacterial metal nanoparticles [211], quaternary ammonium salts [212], and N-halamines [213,214]. Silver has broad-spectrum antimicrobial properties, and it is widely used in wound dressings [7]. Ullah et al. [215] combined PAN nanofibers with good mechanical and thermal properties with silver sulfadiazine (AgSD) with antibacterial properties, and added AgSD by immersion technology to prepare PAN/AgSD nanofiber mats. This nanofiber mat has better antibacterial properties than PAN nanofibers in tensile strength and elongation at break.

Antibiotics began to be put into biomedical use in the 1940s, and since then antibiotic resistance has emerged at an unstoppable and alarming rate [216,217]. Methicillin-resistant Staphylococcus aureus (MRSA) is a very common antibiotic-resistant bacteria, and the recovery rate of wounds infected with MRSA is very low [218], which is very unfavorable for wound healing. There is therefore a need to find new antimicrobial agents to meet the demand for wound dressings in the ever-changing and complex conditions of wounds. Nitric oxide (NO) is a drug-free signaling molecule produced by nitric oxide synthase (NOS) in the body [219]. NO is able to control platelet aggregation, and it is closely related to the production of blood vessels, while a lack of NO can lead to the production of blood clots [220]. NO can play a certain role in maintaining vascular homeostasis, endogenously regulating inflammation [221] and eradicating bacterial infection [222]. Therefore, Workman et al. [201] designed S-nitroso-N-acetylpenicillamine (SNAP)/PAN nanofibers for sustained NO release. SNAP acts as a NO donor attachment, which not only solves the problem of antibiotic-resistant bacteria but also prolongs the use time of wound dressings and reduces the number of dressing changes. When comparing the effects of PAN bandage and ordinary gauze in the wound healing process, we can clearly observe that the ability of PAN to store and transport NO enables the PAN bandage to reduce ineffective NO transport and promote the induction of wound angiogenesis. We all know that in previously underdeveloped medical conditions, wound dressings, such as gauze bandages made of dry cotton, tended to stick to the wound, leading to scab formation and bacterial infection; and, none of the conventional wound dressings have the conditions to continuously provide the wound with active ingredients that promote wound healing. The production of this new type of bandage can better replace traditional wound dressings, and just like a normal gauze bandage, the bandage is easy to apply, and the patient can manage the wound himself at home.

### 5.2. Strategies to Compose Composite Structures from Monolithic Nanofibers in PAN Electrospun Fiber Systems for Wound Dressings

**Figure 9:**
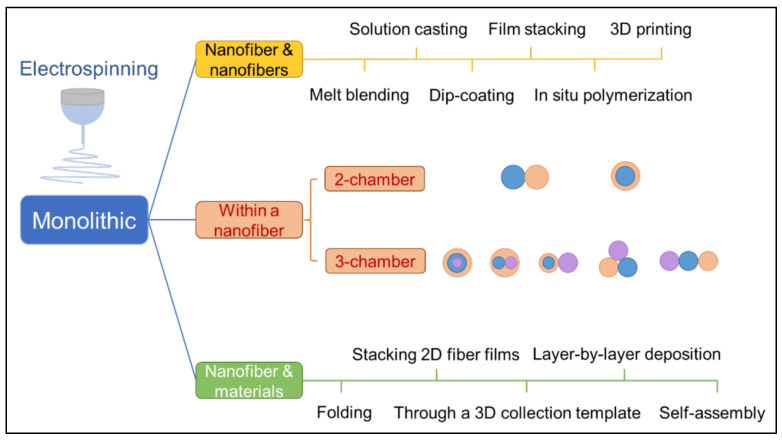
The method of composite nanostructures was developed from uniform nanofibers by improving the electrospinning process, changing the spinneret structure of electrospinning, and combining electrospinning nanofibers with other materials.

The monolithic nanofibers obtained by traditional electrospinning technology have a single structure. In the current research, there have been many methods to develop composite nanostructures from uniform nanofibers, as shown in Figure 9. These methods have also been put into certain applications in research and applications of PAN-based nanofibers as wound dressings. The following will describe in detail from the research of five different methods.

The improvement of blend electrospinning compared to traditional uniaxial electrospinning makes it unique in wound dressing applications. The PAN nanofibers treated with synthetic cationic antibacterial agent quaternary ammonium salt (QAS) exhibited enhanced antibacterial effect. When the QAS is in contact with the cell surface, the electrostatic interaction between positive and negative charges allows the alkyl groups in the QAS to enter the interior of the bacterium and destroy its cell wall and membrane. Bacteria undergo contact death with a loss of cytoplasmic components. Zhang et al. [212] used the antibacterial properties of QAS to blend PAN after hydrolysis and QAS-octadecyltrimethylammonium chloride (STAC) treatment and PCL to obtain quaternary ammonium salt-modified PAN/PCL electrospun nanofibers. Hydrolysis modification weakened the original mechanical properties of PAN nanofibers. The morphology of PAN-STAC nanofibers was observed, and the fiber diameters were not uniform. The nanofibers obtained by blending with PCL improved this problem, with an average diameter of 279 ± 52 nm, the pore size was small, the tensile strength was 3.04 Mpa, and the elongation at break was 162.0%. The release profile of STAC also showed that STAC was able to strongly bind to hydrolyzed PAN.

**Figure 10:**
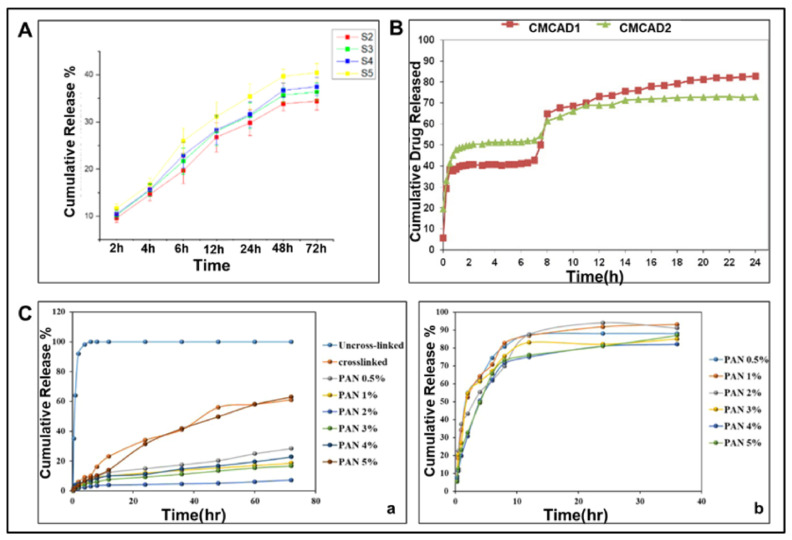
Comparison of drug release profiles from single-drug PAN-based nanofibers, PVA-based nanofibers, and dual-drug PVA/PAN nanofibers with a core-shell structure. (**A**) Drug release profiles of DLF-loaded PAN nanofibers [192] (S2–S5, DLF content of 3, 4, 5, 6 wt% PAN nanofibers, respectively); (**B**) Drug release profiles of DSs-loaded PVA nanofibers, Reprinted with permission from [227] copyright 2016, Taylor & Francis. (**C**) Drug release profiles of PVA-DSs/PAN-GENs core-shell nanofibers [195]. (**a**) Drug release profiles of core DSs; (**b**) drug release profiles of shell GENs.

Wound healing is a very complex process, and the targeted release of multiple drugs in the wound microenvironment can bring great benefits to wound healing in the short term. In order to bring about faster wound healing, the synergistic effect of multiple drugs with a different efficacy are often required. However, uniaxial electrospun fibers limit the release of multiple drugs to specific targets [223]. Furthermore, PAN is mainly soluble in some organic and inorganic solvents, and it has low solubility in water. In this case, PAN nanofibers still have the problem of not being able to select water-soluble drugs for specific wounds [224]. In response to the above two problems, nanofibers with a core-shell structure stand out as a promising dual drug carrier [225,226]. Kharaghani et al. [195] developed a core-shell nanofiber with PVA as the core and PAN as the shell by impregnation method, diclofenac sodium salt (DSs) and gentamicin sulfate (GENs) were added into the core and shell nanofibers, respectively. The thickness of the outer fiber layer prolongs the release of the core DSs, and the release is extended for 48 h after the release of the outer layer GENs. This structure meets the needs of a local dual drug delivery system for wound dressings. Compared with the release profiles and time of DSs in previous studies [192,195,227], the drug release profiles in Figure 10 show the nanofibers with core-shell structure extended the core drug release time from 24 h to 60 h, while the outer layer drug release was not affected. The core-shell structure has made a great contribution to the biphasic release of drugs and has received a lot of attention [228], and it needs to achieve far more than drug release for an ideal wound dressing, which also promotes more development of multi-structured electrospun nanofibers.

**Figure 11:**
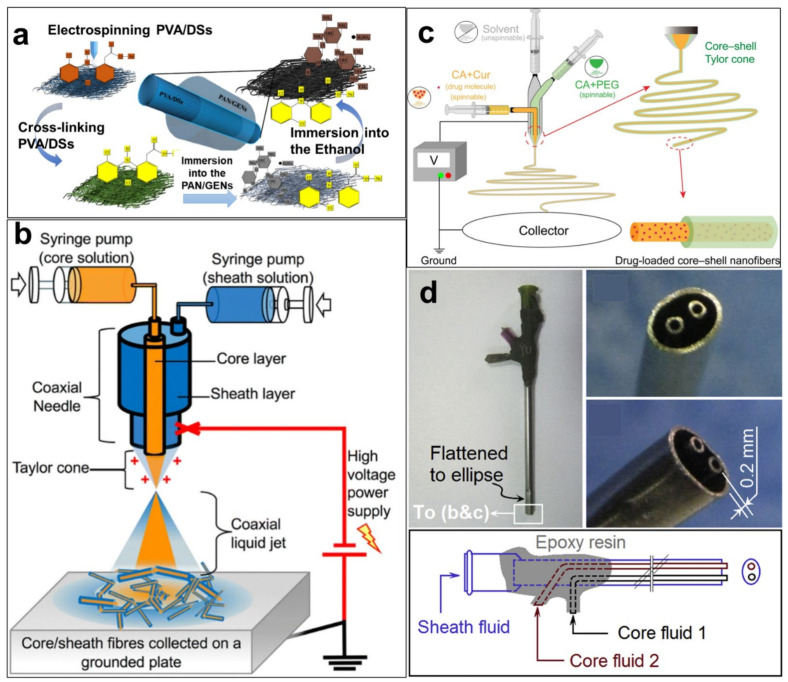
Different preparation methods for wound dressings with core-shell structure. (**a**) Electrospinning and dipping method [195]; (**b**) Coaxial electrospinning method, Reprinted with permission from [132] copyright 2020, Springernature. (**c**) Modified triaxial electrospinning method [131]; (**d**) New trifluid electrospinning method, Reprinted with permission from [117] copyright 2020, Elsevier.Similar to the core-shell structure, the nanofibers with a Janus structure can also play a role in the coexistence of two substances with different properties in the same system [230]. Like PAN nanofiber wound dressings, the existing single-layer wound dressings can quickly absorb the exudate from the wound and bring a moist environment to the wound, but there are certain drawbacks. Such a wound dressing needs to be replaced after a certain period of failure, otherwise the excess exudate on the dressing will complicate the wound dressing process. However, the dressing has been adhered to the wound at this time, and in this case, frequent dressing changes will definitely bring secondary damage to the wound. The materials with a Janus structure can be exactly used to solve this problem. Zhang et al. [231] developed a multi-functional Janus electrospun nanofiber based on polylactide(PLA)/PAN and devoted themselves to the management and real-time monitoring of wound biofluid exudation. Hydrophobic PLA nanofibers can block external bacteria and liquids but do not have the ability to remove biological fluids from wounds. In this study, the researchers constructed multi-functional fiber membranes by spinning a layer of hydrophilic polystyrene pyridine(PSP)/PAN nanofibers on hydrophobic nanofibers. The results showed that the Janus electrospun nanofibers have the ability to unidirectionally drain excess liquid loaded by the dressing and prevent the wet dressing carrying excess liquid from becoming a source of contamination to contaminate the wound environment.

In addition to the above-mentioned combination of electrospinning technology and dipping method [195] to obtain nanofibers with core-shell structure, it can also be prepared by coaxial electrospinning method [229]. The schematic diagrams of the two methods are shown in Figure 11. Coaxial electrospinning is one of the most commonly used methods for core-shell nanofibers, and Han et al. [229] constructed a bifunctional drug delivery system through this technique. In this composite structure, PAN and silk fibroin peptide (SFP) are used as the sheath layer, and PAN and ciprofloxacin (CIP) are used as the core layer. The combination of hydrophilic protein SFP and PAN enhances the spinnability of SFP and brings the ability of PAN to release glycine, which can be used to reduce gastrointestinal adverse reactions, and CIP is a drug that can be absorbed by the stomach. The combination of the two enables the core-shell nanofibers to be used in the treatment of gastrointestinal diseases.

According to existing reports, there is also a layer-by-layer composite nanofiber, which combines two substances with different properties to coexist in the same system to play a role. As we all know, hydrophilic nanofibers can quickly absorb the exudate from the wound and keep the wound environment free from contamination. However, this dressing needs to be changed in time, and it is easy to stick and bring a moist environment to the wound. Hydrophobic nanofibers are just the opposite, so this layer-by-layer nanofiber wound dressing can effectively solve this problem. Zhang et al. [231] developed a PSP/PAN/oxacillin(OXA)/α-K_6_P_2_W_18_O_62_·14H_2_O(P_2_W_18_)/PLA nanofiber wound dressing; the hydrophobicity layer of OXA/P_2_W_18_/PLA nanofibers was used to contact the wound and the hydrophilic layer of PSP/PAN nanofibers outwards. The difference in the hydrophilicity and hydrophobicity of the two layers of fibers made the excess exudate from the wound unidirectionally discharged to the PSP/PAN nanofibers. In this way, while ensuring that the wound environment is not polluted, it can also block external bacteria and liquids, reduce the number of wound dressing changes, and perform exudation management and real-time monitoring of wound exudates.

**Figure 12:**
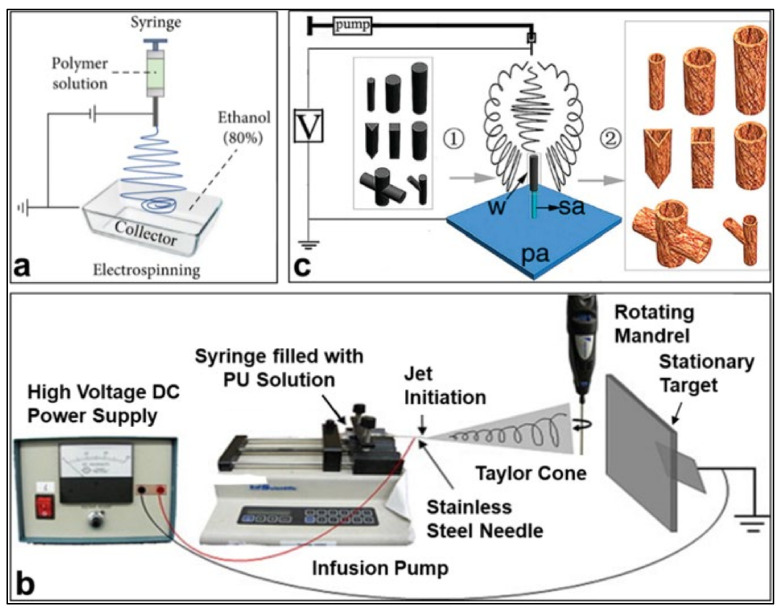
Comparison of devices for forming nanofibers with 3D structures by liquid-assisted collection and template-assisted collection. (**a**) Liquid collector [186]; (**b**) Rotary collector, Reprinted with permission from [233] copyright 2010, Elsevier. (**c**) 3D cylindrical collector, Reprinted with permission from [234], copyright 2008, American Chemical Society.

Compared with one-dimensional (1D) and two-dimensional (2D) nanostructures, three-dimensional (3D) nanostructures can provide better connections and can have a wider application potential [232]. In the electrospinning technology, we complicate the form of the planar collector to form nanofibers with 3D structures [186,233,234] through liquid-assisted collection (as shown in Figure 12a) and template-assisted collection (as shown in Figure 12b,c). However, liquid-assisted collection differs from template-assisted collection in that it forms 3D structures at the microscopic level, with the purpose of expanding the spacing between fibers. Yang et al. [186] fabricated a 3D-structured PAN nanofiber sponge with the aid of a liquid-assisted collector, and its fiber spacing was expanded from 5 µm in 2D fibrous membranes to 15–20 µm.

### 5.3. Wound Healing Effect of Different PAN-Based Nanofiber Wound Dressings

**Figure 13:**
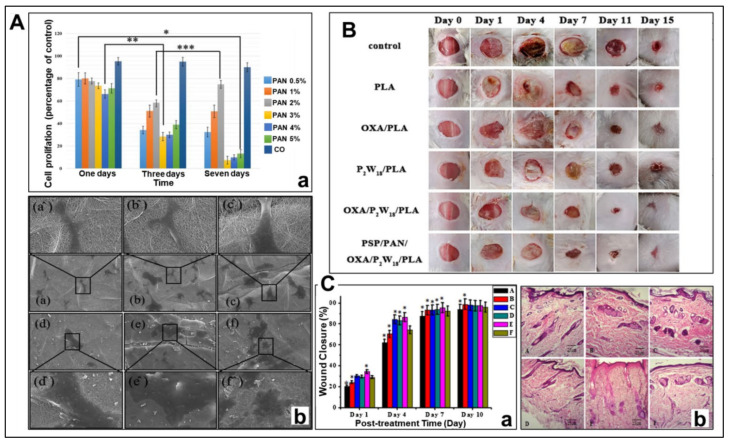
Effect chart of different wound dressings. (**A**) In vitro cell proliferation and cell adhesion experiments of PVA-DSs/PAN-GENs core-shell nanofibers [195]. (**a**) Cell proliferation of different samples on days 1, 3, and 7; (**b**) Cell adhesion and cell migration of different samples (**a**–**f**, PVA-DSs/PAN-GENs core-shell nanofibers with 0.5, 1, 2, 3, 4, and 5% *w/w* PAN/GENs solutions, respectively); (**B**) Wound healing using PSP/PAN/OXA/P_2_W_18_/PLA nanofiber dressing, Reprinted with permission from [231], copyright 2022, American Chemical Society. (**C**) Wound healing study using PAN-moringa extract nanofiber dressing, Reprinted with permission from [196], copyright 2018, American Chemical Society. (**a**) The wound closure rate of PAN-moringa extract nanofiber dressings with different concentrations of Moringa oleifera on the wound for 1, 4, 7, and 10 days; (**b**) Skin tissue images of PAN-moringa extract nanofiber dressings with different concentrations of Moringa oleifera on the 11th day of wound use (Samples **A**–**F** in this study, where **A** is only pure PAN nanofibers, the content of Moringa oleifera extract in **B**–**E** is 0.1, 0.15, 0.2, and 0.5 g, respectively, and **F** is a positive control).

Different wound dressings are suitable for different environments and their mechanisms of action. Figure 13 shows the results of in vitro testing of three different representative PAN-based wound dressings. The wound dressing with core-shell structure constructed with a dual-drug system in Figure 13A reduces inflammation at the wound through the synergistic effect of rapid and sustained release of DSs and GENs [195]. However, the problem of cell adhesion on the fiber surface needs to be further improved to expand its application in some pathological chronic wounds. In Figure 13B, P_2_W_18_ and OXA in the hydrophobic surface fibers of the PSP/PAN/OXA/P_2_W_18_/PLA nanofiber wound dressing that fit the wound work together to enter bacterial cells from the cell wall, directly interfere with cell respiration and kill, having an antibacterial effect. From the experiment in mice, we can observe that compared with other dressings in the control group, the inflammation of the wound with PSP/PAN/OXA/P_2_W_18_/PLA nanofiber wound dressing was significantly reduced on the fourth day, this is closely related to the unidirectional drainage of this layer-by-layer dressing [231]. This significant advantage enables the dressing to be consistently antimicrobial and easily detectable, demonstrating the ability to prevent further infection from transforming into chronic wounds in acute wounds. The wound dressing in Figure 13C utilized Moringa oleifera extract, a plant extract reported to have antioxidant and inflammation-modulating properties [235], which contains flavonoids that lower blood sugar [236]. In this study, the speed of wound closure was also accelerated with the increasing concentration of Moringa oleifera extract, effectively accelerating wound healing [196]. Although these wound dressings have their own effects, they have unique advantages in structure, drug function, and drug transport systems, but for complex chronic wounds there are still certain defects and deficiencies that need to be improved.

### 5.4. Development of Electrospinning Technology in the Application of Wound Dressing

The excellent properties of electrospun nanofibers make them unique in wound dressing application, although their scope for commercial applications is relatively limited. The reason is that electrospinning technology has the following drawbacks: (1) production efficiency is low; (2) equipment cost is high; and (3) compared with the traditional spinning technology, control factors are complicated. However, the ultra-high specific surface area of electrospun nanofibers far exceeds that of traditional spinning technology, which brings the effect of being used as a drug carrier unmatched by other spinning technologies. It is precisely because of this that researchers have made long-term efforts to obtain some relatively satisfactory solutions to the above problems through various technical means [237,238]. For example, the cost can be reduced by self-assembling electrospinning by mastering the assembly and construction technology. It is also possible to study the control factors of electrospinning, quantify the system, predict in advance, and control the influence of different control factors on electrospun fibers within a certain range.

In order to realize the industrialization of electrospinning technology, its low production efficiency is the most urgent problem to be solved. Multi-nozzle side-by-side electrospinning is an effective solution to productivity problems, and converting the collector into a rolling operation of the production line can improve productivity. Wang et al. [239] proposed a method to increase productivity by increasing the number of jets. In their study, an unconstrained spinning geometry was used to replace the presence of needles, while generating multi-parallel fiber jets and avoiding needle clogging caused by larger fiber diameters during melting. Compared with traditional melt electrospinning, the feed rate of this scheme is significantly increased, and various thermoplastic materials such as PAN can be processed into mesoscopic and nanoscale fibers. This considerable productivity is one of the strategies to achieve viable industrial-scale manufacturing.

## 6. Summary and Future Outlook

The recent progress in electrospun medicated PAN nanofibers has been reviewed in this article. Compared with traditional wound dressing, at this stage research on wound dressing has been relatively immature. Wound healing is the purpose, but the purpose is not only wound healing, the physiological and pathological problems of its healing process need to be simultaneously considered. Meanwhile, the issues on how to ensure healing while improving healing speed and how to reduce the damage caused by wounds to the human body are also important for consideration. This article first discusses the far-reaching impact of electrospinning technology in wound dressing research based on the introduction of wound types and healing processes and then the strategies and technological advances in the current study to utilize the performance advantages of PAN to modify PAN nanofibers and load active substances on PAN-based nanofibers to obtain PAN-based nanofibers with complex structures are summarized. Finally, the wound healing effect of the developed PAN-based nanofibers as wound dressings is summarized.

However, our ever-changing needs for wound dressings make the research challenging. The synthetic polymer PAN has good spinnability, and it is easily modified. PAN electrospun nanofibers have a high specific surface area, high porosity and good mechanical properties, and considerable application prospects. They also have good application potential in wound dressings. A focus on PAN nanofiber wound dressing research requires that we first pay attention to the poor biocompatibility of PAN compared with natural polymers and some synthetic polymers and the inherent hydrophobic properties of PAN. Secondly, the effective combination of related therapeutic drugs and various functional particles with PAN-based nanofibers can promote wound healing. However, the wound healing mechanism of some drugs and functional particles is not yet clear, and some potentially bioactive substances remain to be developed; furthermore, the role of a single sheet of PAN nanofibers is limited, and the existing preparation process and fiber structure of PAN-based nanofibers for wound dressings are relatively simple. Based on electrospinning technology, the modification technology can be further combined with the complex structure of nanofibers to enrich its wound healing effect. Finally, with the continuous advancement of technology, it is also worth exploring whether and how PAN-based nanofibers with excellent biological activity and mechanical properties can be produced on a large scale. These problems are not insoluble. Faced with these issues, the related research and results obtained by various researchers on the basis of combining and maintaining the excellent performance of PAN also bring us inspiration for future development.

Based on the above, future research should focus on the large-scale production and improvement of PAN performance; the improvement of PAN-based nanofiber wound dressing preparation strategy and the effect of practical application; and the development of loaded drugs and functional particles. Meanwhile, by changing the structure of the spinning head in electrospinning technology, nanofibers with a Janus structure can be obtained, which have different application effects than the core-shell structure. There is no related research on this structure in the current application of PAN-based nanofiber wound dressings, and the application of this structure can be included in the research in the future. Certainly, tri-axial electrospinning, the combinations of coaxial electrospinning and side-by-side electrospinning, and the combinations of electrospinning with other traditional polymeric treatment methods can bring out even more strategies [240,241,242,243,244]. Beyond that, we can work on developing new smart patches that both treat and monitor wounds. For chronic wounds such as diabetes, the possibility of infection and complications is much greater than that of acute wounds, bringing a burden on the patient’s body and mind. The increase in electrical transport and the construction of cellular communication networks can form a more ideal wound environment and function, putting the development of wound dressings on a new path toward smart multi-functional wound dressings and flexible wearable sensors [245,246]. Undoubtedly, these strategies will further ensure a promising future of PAN nanofiber-based wound dressings for commercial products.

## Figures and Tables

**Table 1:** Some polymers are blended with PAN for electrospinning.

Polymers Blended with PAN	Solvent	Mean Fiber Diameter (nm)	Ref.
Cellulose acetate	DMF	200–500	[172]
Polyaniline	DMF	87–190	[173]
Cellulose acetate butyrate	DMF/acetone	883	[150]
GEL	DMSO	/	[174]
Lignin	DMF	70.77–333.75	[175]
Polyamidoamine	DMF	200–500	[176]
Polymethylhydrosiloxane	DMF	/	[177]
Poly (methyl methacrylate)	DMF	/	[178]
Poly(vinylidene fluoride)	DMF	745–825	[179]
Polysulfone	1-methyl-2-pyrrolidone/DMF	250–500	[180]
Poly(vinylalcohol)	DMSO	/	[181]
β-cyclodextrin	DMF	280–680	[182]

**Table 2:** Drugs used in PAN drug-loaded nanofiber wound dressings.

Drug	Solvent	Advantages	Ref.
Diclofenac sodium	DMF	At 6% density, the *Escherichia coli (E. coli)* and *Staphylococcus aureus (S. aureus)* inhibition zones were shown to be 16 ± 0.46 mm and 15.5 ± 0.28 mm, respectively. DLF-loaded nanofibers showed better cell viability and the dressing still had good biocompatibility.	[192]
Eugenol	/	In vitro antibacterial activity against *C. albicans*. With the effect of analgesic	[193]
Hesperidin	DMF	With the effects of antibacterial, anti-inflammatory, antioxidant, and angiogenic. It has shown in vitro that when a one-centimeter-diameter wound on the back of a rat was treated with Hesperidin-loaded nanofibers, Hesperidin-free nanofibers, and normal saline, the former wound closure was significantly faster than the latter two.	[194]
Gentamine sulfate	DMF	An antibiotic drug. Reduces inflammation and promotes wound regeneration.	[195]
Moringa extract	Ethanol/DMF	It has an effective inhibitory effect on multidrug-resistant methicillin-resistant Staphylococcus aureus. In 16 wt% PAN nanofibers, the higher the concentration of Moringa oleifera extract, the better the antibacterial activity against Escherichia coli and Staphylococcus aureus.	[196]
Tamoxifen	DMF	In clinical medicine, it is used for the prevention and treatment of advanced breast cancer.	[197]
Curcumin	DMF	The hybrid spun fibers containing Cur have enhanced mechanical properties and biocompatibility, and the Cur is distributed within the fibers in an amorphous state.	[198]
Vitamin E acetate	DMF	Fat-soluble antioxidants. Hybrid spun fibers with vitamin E acetate have enhanced mechanical properties and biocompatibility.	[198]

## Data Availability

The data supporting the findings of this manuscript are available from the corresponding authors upon reasonable request.

## Abbreviations

PAN	polyacrylonitrile
*C. albicans*	*Candida albicans*
DMF	N,N-dimethylformamide
DMAc	N,N-dimethylacetamide
DMSO	dimethyl sulfoxide
TETA	triethylenetetramine
BSA	bovine serum albumin
bPEI	branched polyaniline
SA	sodium alginate
CS	chitosan
GEL	gelatin
HA	hyaluronic acid
PVA	polyvinyl alcohol
PAM	polyacrylamide
PEG	polyethylene glycol
PMMA	polymethylmethacrylate
PCL	polycaprolactone
PU	Polyurethane
PANI	polyaniline
*E. coli*	*Escherichia coli*
*S. aureus*	*Staphylococcus aureus*
DLF	diclofenac
MOF	Metal-organic framework
AgSD	silver sulfadiazine
MRSA	Methicillin-resistant Staphylococcus aureus
NO	Nitric oxide
NOS	nitric oxide synthase
SNAP	S-nitroso-N-acetylpenicillamine
QAS	quaternary ammonium salt
STAC	QAS-octadecyltrimethylammonium chloride
DSs	diclofenac sodium salt
GENs	gentamicin sulfate
SFP	silk fibroin peptide
CIP	ciprofloxacin
PLA	polylactide
PSP	polystyrene pyridine
OXA	oxacillin
P_2_W_18_	α-K_6_P_2_W_18_O_62_·14H_2_O
1D	one-dimensional
2D	two-dimensional
3D	three-dimensional
